# Analysis of 5′ Nontranslated Region of Hepatitis A Viral RNA Genotype I from South Korea: Comparison with Disease Severities

**DOI:** 10.1371/journal.pone.0015139

**Published:** 2010-12-28

**Authors:** Tatsuo Kanda, Sook-Hyang Jeong, Fumio Imazeki, Keiichi Fujiwara, Osamu Yokosuka

**Affiliations:** 1 Department of Medicine and Clinical Oncology, Graduate School of Medicine, Chiba University, Chiba, Japan; 2 Department of Internal Medicine, Seoul National University Bundang Hospital, Seongnam-si, Republic of Korea; Charité-University Medicine Berlin, Germany

## Abstract

The aim of the study was to analyze genotype I hepatitis A virus (HAV) 5′ nontranslated region (NTR) sequences from a recent outbreak in South Korea and compare them with reported sequences from Japan. We collected a total of 54 acute hepatitis A patients' sera from HAV genotype I [27 severe disease (prothrombin time INR≥1.50) and 27 mild hepatitis (prothrombin time INR <1.00)], performed nested RT-PCR of 5′ NTR of HAV directly sequenced from PCR products (∼300 bp), and compared them with each other. We could detect HAV 5′NTR sequences in 19 of the 54 (35.1%) cases [12 of 27 severe cases (44.4%) and 7 of 27 self-limited cases (25.9%)], all of which were subgenotype IA. Sequence analysis revealed that sequences of severe disease had 93.6%–99.0% homology and of self-limited disease 94.3%–98.6% homology, compared to subgenotype IA HAV GBM wild-type IA sequence. In this study, confirmation of the 5′NTR sequence differences between severe disease and mild disease was not carried out. Comparison with Japanese HAV A10 revealed ^222^C to G or T substitution in 8/12 cases of severe disease and ^222^C to G or T and ^392^G to A substitutions in 5/7 and 4/7 cases of mild disease, respectively, although the nucleotide sequences in this study showed high homology (93.6%–100%). In conclusion, HAV 5′NTR subgenotype IA from Korea had relatively high homology to Japanese sequences previously reported from Japan, and this region would be considered one of the antiviral targets. Further studies will be needed.

## Introduction

Although hepatitis A vaccination is highly effective, providing herd protection and decreasing mortality and morbidity related to the hepatitis A virus (HAV) [1]–[3], HAV is still a common cause of hepatitis reportedly leading to occasional lethal acute liver failure in many countries of the world [4]–[7]. Recently, a rise in the frequency of hepatitis A outbreaks was observed in South Korea, which lies adjacent to Japan, while the number of adult hepatitis A cases in Japan has been progressively decreasing during the last several years. There is a concern regarding a possible HAV epidemic in Japan in the near future, as universal vaccination against hepatitis A is not performed in this country.

HAV is a member of the genus *Hepatovirus* in the *Picornaviridae* family, and has a positive-sense single-stranded RNA genome approximately 7.5 kb in length [8]. The genome codes a large open reading frame (ORF), which is flanked by 5′ nontranslated region (5′NTR) and 3′NTR. The downstream part of 5′NTR represents the internal ribosomal entry site (IRES), which mediates cap-independent translation initiation and is important for HAV replication [9], [10]. 5′NTR of HAV is also known as one of the most highly conserved in the HAV genome sequences, making this region one of the likely candidates for antiviral targets [9], [11]. It was reported that nucleotide variations in the central portion of 5′NTR of HAV may influence the severity of hepatitis A [12].

Human HAV strains can be grouped into four genotypes (I, II, III and IV) and unique simian strains belong to three additional genotypes (IV, V and VI). Between each of these genotypes, the nucleotide sequence varies by 15–20% of the base positions in the P1 region [13]. Genotype I is the most abundant type worldwide, and genotype IA in particular has been reported from North America, Korea, China, Japan and Thailand [14].

The aim of this study is to characterize the recent HAV genotype I 5′NTR sequences in Korea, to compare them with those reported from Japan and to clarify this region as a target candidate for anti-HAV drugs.

## Materials and Methods

### Patients

Fifty-four patients infected with HAV subgenotypes IA and IB were included in this study. Serum samples were collected at four hospitals located in the Seongnam city area, near Seoul, South Korea. Our study was approved by the Seoul National University Bundang Hospital Institutional Review Board (IRB), and we obtained written informed consent from every patient enrolled during Sep 2008 to Aug 2008. We collected serum or plasma samples immediately after hospital admission, and they were stored at −70°C. The 54 patients comprised 27 with severe disease, defined as prolonged prothrombin time [international normalized ratio (INR) > or  = 1.5] and 27 with mild disease: self-limited acute hepatitis in this study (Table S1A & S1B).

### Primers for PCR and Direct Sequencing

For amplification of HAV sequences and bidirectional direct sequencing of the amplified segments, we prepared several primers for PCR and sequencing as previously described [12]. These primers were prepared with the sequence reported by Cohen et al [8].

### Detection of Hepatitis A Virus RNA in Serum

RNA was extracted from sera using the acid guanidinium-phenol-chloroform method. Reverse transcription was performed with HAV genome specific antisense primer (5′-AGTACCTCAGAGGCAAACAC-3′) as previously described [12].

In the first round PCR, 1 µl of 20 µl of the cDNA solution was used. The first round PCR was performed with 50 µl of reaction mixture containing 25 pmol of outer antisense primer (5′-AGTACCTCAGAGGCAAACAC-3′) and sense primer (5′-TCTTGGAAGTCCATGGTGAG-3′), 200 µM of each dNTP, 50 mM KCl, 10 mM Tris HCL (pH 8.3), 1.5 mM MgCl_2_, 0.001% gelatin, and 2.5 units of Ex Taq polymerase (Takara Bio Inc., Ohtsu, Shiga, Japan). Amplification conditions consisted of 35 cycles of 95°C for one minute, 50°C for one minute, and 72°C for one minute, and 1 µl of the first round product was used for the second round of PCR with the same PCR mixture, except 1.0 µM of inner sense primer (5′-GGGACTTGATACCTCACCGC-3′) and antisense primer (5′-CCACATAAGGCCCCAAAGAA-3′) were used. Amplification conditions for the second round were the same as those for the first round. The second-round PCR products (6 µl) were analyzed by 8% polyacrylamide gel electrophoresis, stained with SyBr green (Takara), and visualized by UV transillumination. In all experiments, the negative samples showed negative results for HAV RNA. HAV genotypes were determined by previously described methods based on the VP1-P2A region [14].

### Direct Sequencing of HAV cDNA Fragments

To prepare the sequence template (nucleotides 75-638 of 5′NTR of HAV), PCR products were treated with ExoSAP-ITR (Affymetrix, Inc., Santa Clara, CA), and then sequenced using a BigDye(R) Terminator v3.1 Cycle Sequencing Kit (Life Technologies, Tokyo, Japan). Sequences were analyzed using Applied Biosystems 3730xl (Life Technologies).

### Nucleotide Sequence Accession Numbers

The nucleotide sequence data reported in this article will appear in GenBank nucleotide sequence databases with accession numbers AB571027 to AB571045.

### Phylogenetic Analysis

To examine the heterogeneity of the viral sequences obtained, a phylogenetic tree was constructed using the neighbor joining methods. To confirm the reliability of the phylogenetic tree, bootstrap resampling tests were performed 10,000 times. These analyses were conducted with the Genetyx-WIN program, version 10 (Software Development, Tokyo, Japan).

### Statistical analysis

Differences in proportions among the groups were compared by Fisher's exact probability test, Student's t test and Welch's t test.

## Results

### Clinical Features of Patients with Acute Hepatitis A Genotype 1 in Korea

Characteristics of these patients at admission are summarized in Table S1. There were no differences in age and gender ratio between the severe and mild disease groups. Mean age of the severe and mild disease groups was 32.1±6.1 and 32.6±5.8 years, respectively. Male gender was dominant in both groups (male/female: 19/8 and 18/9 in the severe and mild disease groups, respectively). Almost all patients of both groups were subgenotype IA, with only two and one being subgenotype IB in the severe and mild disease groups, respectively.

### Sequence Analysis of Korean Isolates

Although the VP1/2A region could be detected in the same serum or stool samples of the same patients, we could detect HAV 5′NTR sequences in 19 of the 54 (35.1%) cases [12 of 27 severe cases (44.4%) and 7 of 27 self-limited cases (25.9%)] by reverse-transcription-nested PCR. All these sequences were subgenotype IA. Then we performed further sequence analysis in these 19 patients by the methods of Fujiwara et al [12]. Japanese studies showed that fewer nucleotide variations were found between nucleotides 200 and 500 of 5′NTR in cases of fulminant hepatitis and severe acute hepatitis than in cases of self-limited acute hepatitis [12]. We thusly performed sequence analysis of the region between nucleotides 200 and 500.

**Figure 1:**
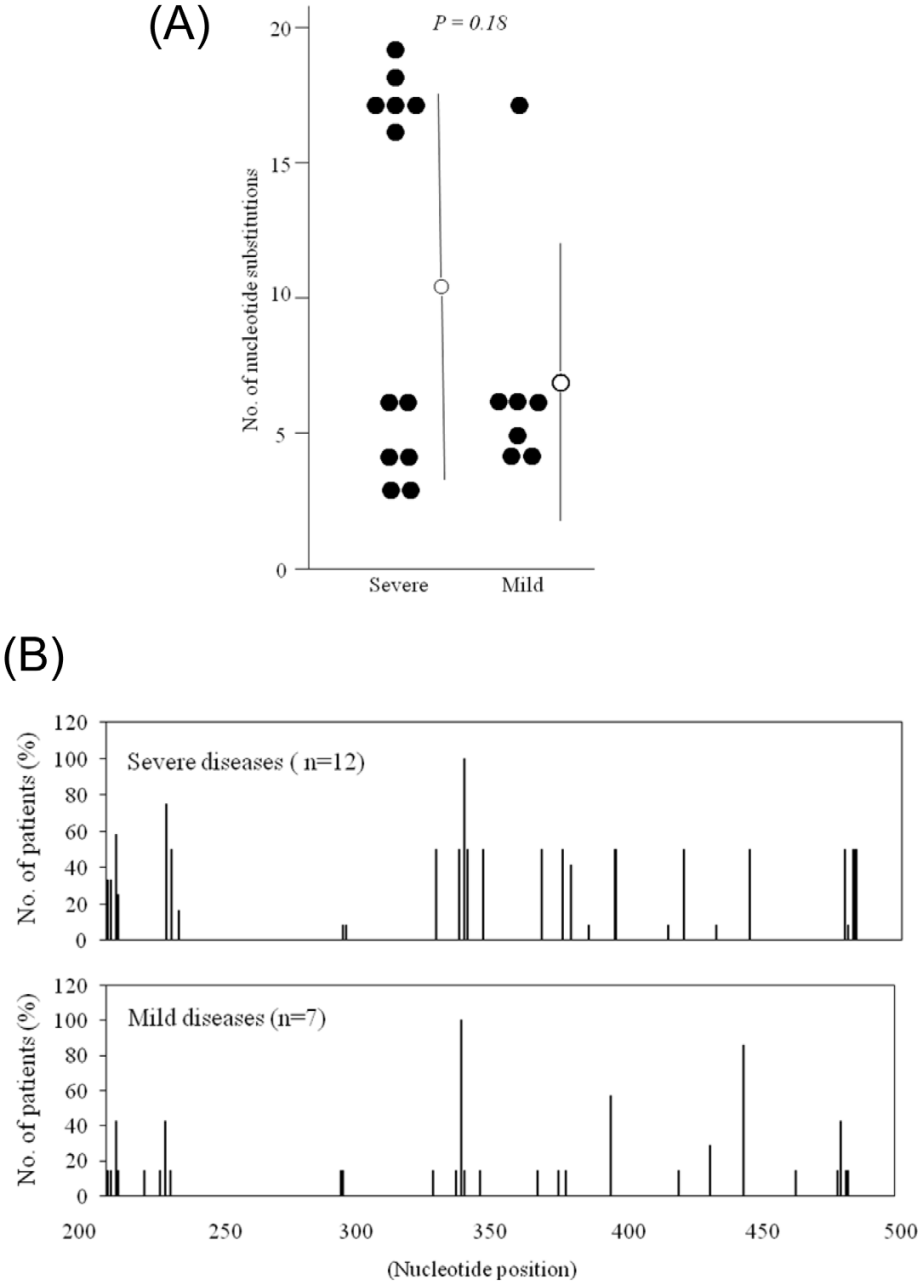
Disease severity and nucleotide substitutions in HAV IRES when compared with HAV GBM. (**A**) Number of nucleotide substitutions between nucleotides 200 and 500. Nucleotide sequences were compared with HAV GBM/WT RNA (X75215) [15]. Bars represent mean (SD). Severe, severe disease; Mild, mild disease. (**B**) Distribution of nucleotide substitutions between nucleotides 200 and 500 of the 5′ non-translated region. Bars indicate the percentage of cases with substitutions at each nucleotide position.

Sequences between nucleotides 200 and 500 were then compared with the wild-type HAV GBM/WT RNA (X75215) [15]. The nucleotide sequence identities of 5′NTR from severe and mild cases ranged from 93.6% to 99.0% and from 94.3% to 98.6%, respectively, compared with wild-type HAV GBM sequence. The distribution of nucleotide variations is shown in Table S2A & S2B. Sequences from cases of severe and mild diseases were mostly similar. Although there was no statistical significance, ^214^C, ^220^T and ^464^T were found in one case each of the mild disease group (Table S2B). On the other hand, ^227^deletion of nucleotide and ^382^A, respectively, were found in two and one cases of the severe disease group (Table S2A). The number of nucleotide substitutions is shown in Figure 1A & 1B. The average number of substitutions between nucleotides 200 and 500 was 10.8 (6.8) [mean (SD)] per case in severe disease and 6.8 (4.5) in mild disease. Differences between severe and mild cases were not statistically significant. We could not construct a phylogenetic tree using these sequences (data not shown).

### Comparison to Japanese HAV Sequences Reported from 1984 to 1999

5′NTR of HAV possesses a secondary structure including stems and loops, functions as an IRES, and plays an important role in translation and replication of this virus [9], [16]. There are six domains in IRES, which is located between nucleotides 151 and 734. Portions of domains III and IV are present between nucleotides 200 and 500. Domain III is located between nucleotides 99 and 323, and domain IV is located between nucleotides 324 and 586. The region between nucleotides 203 and 250 is particularly pyrimidine-rich. To examine the homology with the HAV sequences from Japan reported by Fujiwara et al. [12], we compared the sequences from nucleotides 200 to 500 with A10 (AB045328) from Japan [12]. The nucleotide sequence identities of 5′NTR from severe and mild disease groups ranged from 94.3% to 99.6% and from 93.6% to 100%, respectively, compared with the HAV A10 sequence [12] (Table S3A & S3B). In the Korean group, we found ^222^C to G or T substitution in 8/12 cases of severe disease and ^222^C to G or T and ^392^G to A substitutions in 5/7 and 4/7 cases of mild disease, respectively.

**Figure 2:**
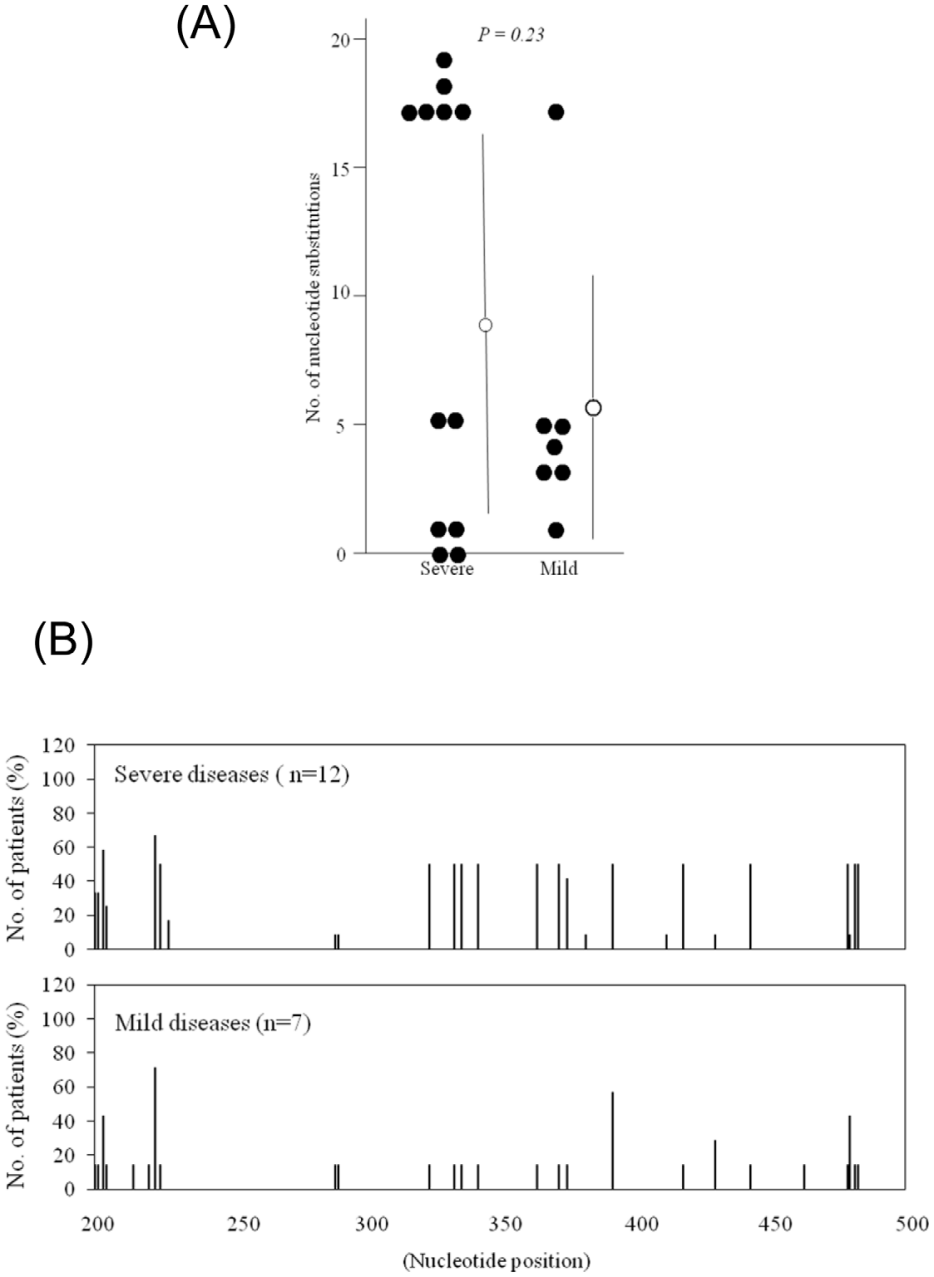
Disease severity and nucleotide substitutions in HAV IRES when compared with HAV A10. (**A**) Number of nucleotide substitutions between nucleotides 200 and 500 Nucleotide sequences were compared with A10 (AB045328) from Japan [12]. Bars represent mean (SD). Severe, severe disease; Mild, mild disease. (**B**) Distribution of nucleotide substitutions between nucleotides 200 and 500 of the 5′ non-translated region. Bars indicate the percentage of cases with substitutions at each nucleotide position.

The number of nucleotide substitutions is shown in Figure 2A & 2B, with the average number between nucleotides 200 and 500 being 9.7 (8.2) [mean (SD)] per case in severe disease and 5.4 (5.2) in mild disease. Again, differences between severe and mild cases were not statistically significant.

## Discussion

The number of adult hepatitis A cases has been progressively increasing during the last several years in Korea [6], [14]. In Japan, on the other hand, the number of patients with sporadic type A hepatitis has recently been on the decrease. In the 9 years from 1999 inclusive, 763, 381, 491, 502, 303, 139, 170, 320 and 157 hepatitis A cases were reported to the Infectious Disease Surveillance Center, National Institute of Infectious Diseases, Tokyo, Japan (www.nih.go.jp). Japan lies adjacent to Korea, separated by the Sea of Japan. The two countries have some cultural similarities. In Japan, there is no universal vaccination program against hepatitis A and hepatitis B. These circumstances have raised concerns about a possible HAV epidemic in Japan. We then analyzed HAV genome sequences from Korea and compared them with the reported sequences from Japan over the past several years.

In the present study, as most of the HAV strains belonged to subgenotype IA in Korea [14], we chose only genotype I patients for analysis. Among 54 HAV IgM positive sera, 35.1% (n = 19) were positive for HAV RNA by nested RT-PCR for 5′NTR. All these strains belonged to subgenotype IA. We tried to perform phylogenetic tree analysis, but these 19 strains formed a single cluster to which almost all Japanese sequences reported by Fujiwara et al [12] belonged (data not shown). Fujiwara et al [12] found an association between the severity of hepatitis A and nucleotide variations in 5′NTR of Japanese HAV RNA. In the present study, we did not confirm 5′NTR sequence differences between severe disease and mild disease.

The age of HAV sequence-analyzed patients in the present study was 30.5±5.9 and 31.4±5.0 years, respectively, in severe and mild diseases. The gender of HAV sequence-analyzed patients was male-dominant (male/female: 8/4 and 6/1 in the severe disease and mild disease groups, respectively). In the study by Fujiwara et al [12], the patients were also male-dominant, but their age with fulminant hepatitis and severe acute hepatitis (43.1±14.4 year, *P* = 0.010 and 41.6±12.6, *P* = 0.010, respectively) was significantly higher than the age of severe-disease patients. On the other hand, the age of their patients with self-limited acute hepatitis was similar to that of our mild-disease patients. We defined patients with prothrombin time INR ≥1.50 as severe hepatitis in this study, whereas Fujiwara et al [12] defined patients with prothrombin time of less than 40% as severe hepatitis with (fulminant hepatitis) or without encephalopathy (severe acute hepatitis).

In Japan, similar to the situation in Korea [6], young adults seem not to have protective antibody against HAV, and so it appears that hepatitis A cases can be expected to increase in the near future.

A previous study showed that the 5′ border of IRES is located between nucleotides 151 and 257, while the 3′ border extends to the 3′ end of 5′NTR, between nucleotide 695 and the first initiation codon at 735 [17]. ^222^C to G or T substitution was located on the loop structure at domain IIIa of HAV IRES. A previous Japanese study showed that nucleotide 225 substitutions occurred in 80% of the sequences around nucleotide position 222 [12]. ^392^G to A substitution located at domain IV of HAV IRES was observed in 64.2% (9/14) of the Korean HAV sequences. Fujiwara et al [12] also reported that substitutions at nucleotide 391 were seen in 32% of Japanese HAV patients. It is possible that these substitutions were non-specific mutations.

In conclusion, HAV 5′NTR subgenotype IA from Korea had relatively high homology to the Japanese sequences previously reported, and this region may represent a viable antiviral target. In Japan, as in Korea, the introduction of childhood vaccination and catch-up vaccination for adolescents and young adults should be considered.

## Supporting Information

Table S1 **Patient Characteristics.** (A) Severe disease, (B) Mild disease.
(DOC)

Table S2 **Comparison of the nucleotide sequences of the HAV 5′ non-translated region with GBM.** (A) Severe disease, (B) Mild disease. The consensus sequence for HAV GBM/WT RNA (X75215) [15] is shown on the top. Dots indicate conserved nucleotides; differences are shown by the appropriate single letter nucleotide. -, deletion mutant.
(DOC)

Table S3 **Comparison of the nucleotide sequences of the HAV 5′ non-translated region with GBM.** (A) Severe disease, (B) Mild disease. The consensus sequence for A10 (AB045328) from Japan [12] is shown on the top. Dots indicate conserved nucleotides; differences are shown by the appropriate single letter nucleotide. -, deletion mutant.
(DOC)
