# Supplementary material for: Analysis of 5′ Nontranslated Region of Hepatitis A Viral RNA Genotype I from South Korea: Comparison with Disease Severities
Source: PLoS One. 2010 Dec 28;5(12):e15139. doi: 10.1371/journal.pone.0015139 (PMC3010980; doi:10.1371/journal.pone.0015139)
Supplement: Table S1 — Patient Characteristics. (A) Severe disease, (B) Mild disease. (DOC) [file pone.0015139.s001.doc]

**Table S1 Patient Characteristics**

1. **Severe disease**

| Patient | Age/Gender | PT, INR | Genotype | 5'NTR PCR |
| --- | --- | --- | --- | --- |
|  |  |  |  |  |
| 1 | 45/Male | 4.85 | 1B | negative |
| 2 | 39/Male | 2.65 | 1A | negative |
| 3 | 27/Male | 2.4 | 1A | positive |
| 4 | 37/Male | 2.13 | 1A | negative |
| 5 | 36/Male | 2.09 | 1A | negative |
| 6 | 24/Male | 2.02 | 1A | positive |
| 7 | 30/Male | 1.94 | 1A | positive |
| 8 | 34/Female | 1.91 | 1A | negative |
| 9 | 28/Female | 1.82 | 1A | positive |
| 10 | 22/Male | 1.75 | 1B | negative |
| 11 | 33/Female | 1.7 | 1A | positive |
| 12 | 27/Male | 1.68 | 1A | negative |
| 13 | 30/Male | 1.67 | 1A | negative |
| 14 | 31/Female | 1.65 | 1A | negative |
| 15 | 45/Male | 1.64 | 1A | positive |
| 16 | 32/Male | 1.62 | 1A | negative |
| 17 | 31/Male | 1.62 | 1A | positive |
| 18 | 39/Male | 1.6 | 1A | negative |
| 19 | 26/Male | 1.6 | 1A | positive |
| 20 | 25/Male | 1.59 | 1A | negative |
| 21 | 36/Male | 1.54 | 1A | positive |
| 22 | 32/Female | 1.53 | 1A | positive |
| 23 | 29/Female | 1.51 | 1A | negative |
| 24 | 23/Female | 1.51 | 1A | positive |
| 25 | 31/Male | 1.5 | 1A | positive |
| 26 | 38/Female | 1.49 | 1A | negative |
| 27 | 37/Male | 2.06 | 1A | negative |

1. **Mild disease**

| Patient | Age/Gender | PT, INR | Genotype | 5'NTR PCR |
| --- | --- | --- | --- | --- |
|  |  |  |  |  |
| 28 | 35/Male | 0.88 | 1A | negative |
| 29 | 36/Male | 0.9 | 1A | negative |
| 30 | 36/Male | 0.9 | 1A | negative |
| 31 | 26/Male | 0.91 | 1A | negative |
| 32 | 27/Female | 0.91 | 1A | negative |
| 33 | 34/Female | 0.92 | 1A | negative |
| 34 | 35/Male | 0.92 | 1A | negative |
| 35 | 43/Female | 0.94 | 1A | negative |
| 36 | 24/Male | 0.95 | 1A | negative |
| 37 | 41/Female | 0.96 | 1A | negative |
| 38 | 34/Female | 0.96 | 1A | negative |
| 39 | 34/Female | 0.97 | 1A | negative |
| 40 | 30/Male | 0.97 | 1A | negative |
| 41 | 35/Male | 0.98 | 1A | negative |
| 42 | 31/Male | 0.98 | 1A | positive |
| 43 | 36/Male | 0.98 | 1A | positive |
| 44 | 34/Male | 0.98 | 1A | positive |
| 45 | 32/Female | 0.98 | 1A | negative |
| 46 | 26/Female | 0.99 | 1A | negative |
| 47 | 33/Male | 0.99 | 1A | negative |
| 48 | 29/Male | 0.99 | 1A | negative |
| 49 | 35/Female | 1 | 1A | positive |
| 50 | 26/Male | 1 | 1A | positive |
| 51 | 35/Male | 1 | 1A | positive |
| 52 | 24/Male | 1 | 1A | negative |
| 53 | 23/Male | 1 | 1A | positive |
| 54 | 47/Male | 1 | 1B | negative |

The consensus sequence for A10 (AB045328) from Japan [12] is shown on the top. Dots indicate conserved nucleotides; differences are shown by the appropriate single letter nucleotide. -, deletion mutant.
