# Supplementary material for: Analysis of 5′ Nontranslated Region of Hepatitis A Viral RNA Genotype I from South Korea: Comparison with Disease Severities
Source: PLoS One. 2010 Dec 28;5(12):e15139. doi: 10.1371/journal.pone.0015139 (PMC3010980; doi:10.1371/journal.pone.0015139)
Supplement: Table S2 — Comparison of the nucleotide sequences of the HAV 5′ non-translated region with GBM. (A) Severe disease, (B) Mild disease. The consensus sequence for HAV GBM/WT RNA (X75215) [15] is shown on the top. Dots indicate conserved nucleotides; differences are shown by the appropriate single letter nucleotide. -, deletion mutant. (DOC) [file pone.0015139.s002.doc]

**Table S2 Comparison of the nucleotide sequences of the HAV 5’ non-translated region with GBM.**

1. **Severe disease**

| Nt. | 200 | 201 | 203 | 204 | 214 | 220 | 222 | 224 | 227 | 289 | 290 | 324 | 333 | 335 | 336 |
| --- | --- | --- | --- | --- | --- | --- | --- | --- | --- | --- | --- | --- | --- | --- | --- |
| GBM | T | C | A | T | T | C | T | - | C | A | T | G | T | A | G |
| Pt. No. |  |  |  |  |  |  |  |  |  |  |  |  |  |  |  |
| 3 | . | . | - | - | . | . | G | T | . | . | . | A | G | G | A |
| 6 | . | . | - | - | . | . | . | T | . | . | . | A | G | G | A |
| 7 | . | . | . | . | . | . | . | . | . | G | C | . | . | G | . |
| 9 | . | . | . | . | . | . | C | . | - | . | . | . | . | G | . |
| 11 | . | . | - | - | . | . | G | T | . | . | . | A | G | G | A |
| 15 | . | . | . | . | . | . | C | . | - | . | . | . | . | G | . |
| 17 | . | . | . | . | . | . | C | . | . | . | . | . | . | G | . |
| 19 | C | A | T | . | . | . | . | . | . | . | . | . | . | G | . |
| 21 | . | . | . | . | . | . | C | . | . | . | . | . | . | G | . |
| 22 | - | - | T | . | . | . | G | T | . | . | . | A | G | G | A |
| 24 | - | - | T | . | . | . | G | T | . | . | . | A | G | G | A |
| 25 | - | - | T | . | . | . | G | T | . | . | . | A | G | G | A |
|  |  |  |  |  |  |  |  |  |  |  |  |  |  |  |  |
| Nt. | 342 | 364 | 372 | 375 | 382 | 392 | 412 | 418 | 430 | 443 | 463 | 479 | 480 | 482 | 483 |
| GBM | G | C | C | C | G | G | C | G | T | T | C | G | T | A | A |
| Pt. No. |  |  |  |  |  |  |  |  |  |  |  |  |  |  |  |
| 3 | A | T | G | T | . | A | . | A | . | . | . | A | . | - | T |
| 6 | A | T | G | T | . | A | . | A | . | . | . | A | . | - | T |
| 7 | . | . | . | . | A | . | . | . | . | C | . | . | C | . | . |
| 9 | . | . | . | . | . | . | . | . | . | C | . | . | . | . | . |
| 11 | A | T | G | T | . | A | . | A | . | . | . | A | . | - | T |
| 15 | . | . | . | . | . | . | . | . | . | C | . | . | . | . | . |
| 17 | . | . | . | . | . | . | . | . | . | C | . | . | . | . | . |
| 19 | . | . | . | . | . | . | . | . | A | C | . | . | . | . | . |
| 21 | . | . | . | . | . | . | . | . | . | C | . | . | . | . | . |
| 22 | A | T | T | . | . | A | . | A | . | . | . | A | . | - | T |
| 24 | A | T | G | T | . | A | T | A | . | . | . | A | . | - | T |
| 25 | A | T | G | T | . | A | . | A | . | . | . | A | . | - | C |

1. **Mild disease**

| Nt. | 200 | 201 | 203 | 204 | 214 | 220 | 222 | 224 | 227 | 289 | 290 | 324 | 333 | 335 | 336 |
| --- | --- | --- | --- | --- | --- | --- | --- | --- | --- | --- | --- | --- | --- | --- | --- |
| GBM | T | C | A | T | T | C | T | - | C | A | T | G | T | A | G |
| Pt. No. |  |  |  |  |  |  |  |  |  |  |  |  |  |  |  |
| 42 | . | . | - | . | . | T | C | . | . | . | . | . | . | G | . |
| 43 | . | . | - | - | . | . | G | T | . | . | . | A | G | G | A |
| 44 | C | A | T | . | . | . | . | . | . | . | . | . | . | G | . |
| 49 | . | . | . | . | . | . | . | . | . | G | C | . | . | G | . |
| 50 | . | . | . | . | . | . | . | . | . | . | . | . | . | G | . |
| 51 | . | . | . | . | C | . | C | . | . | . | . | . | . | G | . |
| 53 | . | . | . | . | . | . | . | . | . | . | . | . | . | G | . |
|  |  |  |  |  |  |  |  |  |  |  |  |  |  |  |  |
| Nt. | 342 | 364 | 372 | 375 | 382 | 392 | 412 | 418 | 430 | 443 | 463 | 479 | 480 | 482 | 483 |
| GBM | G | C | C | C | G | G | C | G | T | T | C | G | T | A | A |
| Pt. No. |  |  |  |  |  |  |  |  |  |  |  |  |  |  |  |
| 42 | . | . | . | . | . | . | . | . | A | C | . | . | . | . | . |
| 43 | A | T | G | T | . | A | . | A | . | . | . | A | . | - | T |
| 44 | . | . | . | . | . | . | . | . | A | C |  | . | . | . | . |
| 49 | . | . | . | . | . | A | . | . | . | C | . | . | C | . | . |
| 50 | . | . | . | . | . | A | . | . | . | C | T | . | C | . | . |
| 51 | . | . | . | . | . | . | . | . | . | C | . | . | . | . | . |
| 53 | . | . | . | . | . | A | . | . | . | C | . | . | C | . | . |

The consensus sequence for HAV GBM/WT RNA (X75215) [15] is shown on the top. Dots indicate conserved nucleotides; differences are shown by the appropriate single letter nucleotide. -, deletion mutant.
