# Supplementary material for: Analysis of 5′ Nontranslated Region of Hepatitis A Viral RNA Genotype I from South Korea: Comparison with Disease Severities
Source: PLoS One. 2010 Dec 28;5(12):e15139. doi: 10.1371/journal.pone.0015139 (PMC3010980; doi:10.1371/journal.pone.0015139)
Supplement: Table S3 — Comparison of the nucleotide sequences of the HAV 5′ non-translated region with GBM. (A) Severe disease, (B) Mild disease. The consensus sequence for A10 (AB045328) from Japan [12] is shown on the top. Dots indicate conserved nucleotides; differences are shown by the appropriate single letter nucleotide. -, deletion mutant. (DOC) [file pone.0015139.s003.doc]

**Table S3 Comparison of the nucleotide sequences of the HAV 5’ non-translated region with GBM.**

1. **Severe disease**

| Nt. | 200 | 201 | 203 | 204 | 214 | 220 | 222 | 224 | 227 | 289 | 290 | 324 | 333 | 336 | 342 |
| --- | --- | --- | --- | --- | --- | --- | --- | --- | --- | --- | --- | --- | --- | --- | --- |
| A10 | T | C | A | T | T | C | C | - | C | A | T | G | T | G | G |
| Pt. No. |  |  |  |  |  |  |  |  |  |  |  |  |  |  |  |
| 3 | . | . | - | - | . | . | G | T | . | . | . | A | G | A | A |
| 6 | . | . | - | - | . | . | T | T | . | . | . | A | G | A | A |
| 7 | . | . | . | . | . | . | T | . | . | G | C | . | . | . | . |
| 9 | . | . | . | . | . | . | . | . | - | . | . | . | . | . | . |
| 11 | . | . | - | - | . | . | G | T | . | . | . | A | G | A | A |
| 15 | . | . | . | . | . | . | . | . | - | . | . | . | . | . | . |
| 17 | . | . | . | . | . | . | . | . | . | . | . | . | . | . | . |
| 19 | C | A | T | . | . | . | T | . | . | . | . | . | . | . | . |
| 21 | . | . | . | . | . | . | . | . | . | . | . | . | . | . | . |
| 22 | - | - | T | . | . | . | G | T | . | . | . | A | G | A | A |
| 24 | - | - | T | . | . | . | G | T | . | . | . | A | G | A | A |
| 25 | - | - | T | . | . | . | G | T | . | . | . | A | G | A | A |
|  |  |  |  |  |  |  |  |  |  |  |  |  |  |  |  |
| Nt. | 364 | 372 | 375 | 382 | 392 | 412 | 418 | 430 | 443 | 463 | 479 | 480 | 482 | 483 |  |
| A10 | C | C | C | G | G | C | G | T | C | C | G | T | A | A |  |
| Pt. No. |  |  |  |  |  |  |  |  |  |  |  |  |  |  |  |
| 3 | T | G | T | . | A | . | A | . | T | . | A | . | - | T |  |
| 6 | T | G | T | . | A | . | A | . | T | . | A | . | - | T |  |
| 7 | . | . | . | A | . | . | . | . | . | . | . | C | . | . |  |
| 9 | . | . | . | . | . | . | . | . | . | . | . | . | . | . |  |
| 11 | T | G | T | . | A | . | A | . | T | . | A | . | - | . |  |
| 15 | . | . | . | . | . | . | . | . | . | . | . | . | . | . |  |
| 17 | . | . | . | . | . | . | . | . | . | . | . | . | . | . |  |
| 19 | . | . | . | . | . | . | . | A | . | . | . | . | . | . |  |
| 21 | . | . | . | . | . | . | . | . | . | . | . | . | . | . |  |
| 22 | T | T | . | . | A | . | A | . | T | . | A | . | - | T |  |
| 24 | T | G | T | . | A | T | A | . | T | . | A | . | - | T |  |
| 25 | T | G | T | . | A | . | A | . | T | . | A | . | - | C |  |

1. **Mild disease**

| Nt. | 200 | 201 | 203 | 204 | 214 | 220 | 222 | 224 | 227 | 289 | 290 | 324 | 333 | 336 | 342 |
| --- | --- | --- | --- | --- | --- | --- | --- | --- | --- | --- | --- | --- | --- | --- | --- |
| A10 | T | C | A | T | T | C | C | - | C | A | T | G | T | G | G |
| Pt. No. |  |  |  |  |  |  |  |  |  |  |  |  |  |  |  |
| 42 | . | . | - | . | . | T | . | . | . | . | . | . | . | . | . |
| 43 | . | . | - | - | . | . | G | T | . | . | . | A | G | A | A |
| 44 | C | A | T | . | . | . | T | . | . | . | . | . | . | . | . |
| 49 | . | . | . | . | . | . | T | . | . | G | C | . | . | . | . |
| 50 | . | . | . | . | . | . | T | . | . | . | . | . | . | . | . |
| 51 | . | . | . | . | C | . | . | . | . | . | . | . | . | . | . |
| 53 | . | . | . | . | . | . | T | . | . | . | . | . | . | . | . |
|  |  |  |  |  |  |  |  |  |  |  |  |  |  |  |  |
| Nt. | 364 | 372 | 375 | 382 | 392 | 412 | 418 | 430 | 443 | 463 | 479 | 480 | 482 | 483 |  |
| A10 | C | C | C | G | G | C | G | T | C | C | G | T | A | A |  |
| Pt. No. |  |  |  |  |  |  |  |  |  |  |  |  |  |  |  |
| 42 | . | . | . | . | . | . | . | A | . | . | . | . | . | . |  |
| 43 | T | G | T | . | A | . | A | . | T | . | A | . | - | T |  |
| 44 | . | . | . | . | . | . | . | A | . | . | . | . | . | . |  |
| 49 | . | . | . | . | A | . | . | . | . | . | . | C | . | . |  |
| 50 | . | . | . | . | A | . | . | . | . | T | . | C | . | . |  |
| 51 | . | . | . | . | . | . | . | . | . | . | . | . | . | . |  |
| 53 | . | . | . | . | A | . | . | . | . | . | . | C | . | . |  |

The consensus sequence for A10 (AB045328) from Japan [12] is shown on the top. Dots indicate conserved nucleotides; differences are shown by the appropriate single letter nucleotide. -, deletion mutant.
